# Eugenol Essential Oil and Nanoemulsion as Antihydatic Agents with Antifibrotic and Immunomodulatory Effects in Cystic Echinococcosis

**DOI:** 10.3390/tropicalmed8050253

**Published:** 2023-04-27

**Authors:** Alzahraa Abdelraouf Ahmad, Maria Naged Maurice, Mohamed El-Salahy M. Monib, Mahmoud Soliman, Sultan S. Al-Thagfan, Enas Abdelhameed Mahmoud Huseein

**Affiliations:** 1 Department of Medical Parasitology, Faculty of Medicine, Assiut University, Assiut 71515, Egypt; 2 Department of Pathology and Clinical Pathology, Faculty of Veterinary Medicine, Assiut University, Assiut 71515, Egypt; 3 Department of Immunology, University of Texas Southwestern Medical Center, Dallas, TX 75235, USA; 4 Department of Clinical and Hospital Pharmacy, College of Pharmacy, Taibah University, Al Madinah Al Munawarah 30001, Saudi Arabia

**Keywords:** cystic echinococcosis, eugenol, eugenol nanoemulsion, cytokine profile, STAT4, GATA3

## Abstract

Conventional scolicidal agents are still unsatisfactory in combating hydatid disease due to their low efficacy and increased drug side effects. Therefore, novel scolicides are required. This study aimed to evaluate the antihydatic and immunomodulatory effects of eugenol essential oil (Eug) and its nanoemulsion (Eug-NE) in cystic echinococcosis (CE). Eug and Eug-NE were administered orally to CE-infected rats and compared to albendazole (ABZ). Hydatid cyst development was assessed based on organ weight and hypertrophy indicators of the infected organs, along with a histopathological and histochemical evaluation of collagen content. The immunomodulatory effects of treatment on CE were evaluated by serum cytokine levels measurement of interferon-γ (IFN-γ) and interleukin (IL)-4 and immunohistochemical (IHC) analysis of signal transducer and activator of transcription 4 (STAT4) and GATA-binding protein 3 (GATA3) markers. Eug-NE was the most effective in reducing the cyst weights, organ weights, and hypertrophy indicators and improving histopathological lesions with reduced collagen content. Eug and Eug-NE significantly increased the IFN-γ levels and decreased the IL-4 levels, while IHC analysis demonstrated a significant reduction in STAT4 and GATA3 expression in all treated groups. Eug and Eug-NE demonstrated antihydatic and preventative effects, with a substantial decrease in liver fibrosis compared to that of ABZ. Besides their promising immunomodulatory effects, their good treatment response suggests their use as alternatives or complementary scolicidal agents in hydatid cyst treatment.

## 1. Introduction

Cystic echinococcosis (CE), a potentially fatal neglected zoonotic disease with substantial socioeconomic and health impacts, is caused by the larvae of *Echinococcus granulosus* [1]. It has a worldwide distribution, with an emerging or reemerging status in several countries [2,3]. CE affects more than 1 million people globally, with annual economic costs of more than $3 billion in terms of management, besides livestock losses [4]. Echinococcosis can affect many organs, but the liver is the most commonly affected organ, where 70% of metacestodes are found in the right lobe, followed by the lungs; both organs account for 90% of the cases [5].

CE can be treated by surgery, puncture, aspiration, injection, reaspiration (or PAIR), and/or chemotherapy, depending on the patient’s condition, location, size, and number of cysts [6,7].

Albendazole (ABZ), a benzimidazole carbamate derivative, is currently the drug of choice for treating CE in humans [8]. However, no complete recovery occurs after treatment with ABZ [9], as evidenced by the recurrence of infection commonly observed after treatment cessation [10]. Additionally, more than 40% of hydatid cysts could be active 2 years after the onset of therapy [11]. In addition, serious adverse side effects were reported, as ABZ should be administered for longer periods and at higher dosages [12]. Therefore, new and more efficient candidate drugs for CE treatment are needed to overcome these deficiencies and maximize their antiparasitic effects [7,13].

Several studies on the effectiveness of numerous essential oils and nanoemulsion preparations against *E. granulosus* protoscoleces indicated that these preparations could be promising scolicidal agents that may help manage CE [14,15,16,17,18].

Eugenol (Eug) is a naturally occurring aromatic phenol essential oil that has been widely documented in the literature for its wide biological activities. Eug essential oil was described as a promising chemotherapeutic agent that has been tested as anti-inflammatory, antioxidant, anticancer, antifibrotic, and with different antimicrobial effects [19,20,21,22]. The antiparasitic properties of Eug were widely investigated against many helminths and protozoan parasites, such as *Fasciola*, *Trichinella spiralis, Schistosoma mansoni, Giardia lamblia, Trypanosoma cruzi,* and *Leishmania donovani*, exhibiting high efficacy with promising lethal effects [23,24,25,26,27,28]. Moreover, nanoemulsions have been recently introduced as suitable carriers for active essential oils owing to their easy preparation, thermodynamic stability, and affordable production. Therefore, several researchers studied their antiparasitic properties; however, the antimicrobial and antiparasitic applications of Eug nanoemulsion were less investigated in only a few studies as an antimicrobial agent and a larvicide [29,30]. In addition, a recent in vitro study has suggested that eugenol (Eug) oil and its nanoemulsion showed promising protoscolicidal properties, with high safety and efficacy [31].

The progression of hydatid disease and the subsequent clinical consequences are mainly dependent on the host’s immune mechanism [32]. Several studies showed that the sensitivity and resistance to hydatidosisis dependent on Th1 or Th2 responses [33]. Additionally, apoptosis and numerous innate immune factors such as neutrophils, macrophages, natural killer (NK) cells, and Toll-like receptors have a crucial role in susceptibility or resistance to hydatid infections; however, their importance is unclear [34].

A Th2-dominant response can also increase susceptibility to infection (active cyst), whereas a Th1 response enhances resistance to infection (inactive cyst) [35]. The balance between Th1 and Th2 immune responses plays a vital role in determining the disease’s outcome. Th1 and Th2 immune responses are regulated by the secretion of specific cytokines, such as interferon-γ (IFN-γ; Th1-derived cytokine) and interleukin (IL)-4 (Th2-derived cytokine), and controlled by specific transcription factors [36].

Cellular transcription factors are recognized by their role in the transduction of cellular differentiation and transmission of many cytokine-mediated signals [37]. Signal transducer and activator of transcription 4 (STAT4) promote the differentiation of Th1 immune cells [38], whereas GATA-binding protein 3 (GATA3) is necessary for the characterization of Th0 cells toward the Th2 cell subtype and suppresses their differentiation toward Th1 [39]. Consequently, medications that modulate the immune response are of great value in changing the course of the disease.

As Eug oil and its nanoemulsion showed a profound scolicidal effect [31], besides its well-known immunostimulatory effect in enhancing the Th1 immune response, which could be protective against CE progression [40], this study was designed to gain insight into the antihydatic effects of Eug oil and its nanoemulsion in a CE murine model, with a particular focus on their immunomodulatory properties.

## 2. Materials and Methods

### 2.1. Chemicals and Reagents

Eug (4-allyl-2-methoxyphenol; commercial-grade; >98%, MW 164.20), sodium citrate, and RPMI-1640 medium were purchased from Sigma-Aldrich Chemical Co. Ltd. (Schnelldorf, Germany). Pure ABZ was donated by EPICO (Cairo, Egypt). Biochemical reagents (Tween 20, dimethyl sulfoxide, and phosphate-buffered saline) were obtained from Alpha Chemical (Cairo, Egypt). Hanks’ balanced salt solution (HBSS) was purchased from Biowest Biotechnology Co. (Rue de la Caille, Nuaillé, France). STAT4 and GATA3 rabbit polyclonal antibodies were purchased from ABclonal (Woburn, MA, USA). Enzyme-linked immunosorbent assay (ELISA) kits for IL4 and IFN-γ were purchased from OriGene^®^ (Rockville, MD, USA).

### 2.2. Preparation and Characterization of 10% Eug Nanoemulsion (Eug-NE)

Eug-NE was prepared and characterized using the high-pressure homogenization method The preparation technique and characterization methods for the assessment of Eug-NE were described in detail in a previous in vitro study published by the authors [31]. Briefly, the average particle size and the polydispersity index (PDI) values of Eug-NE were 65 ± 11.11 nm and 1.00, respectively, as determined by the Zetasizer. Light scattering was performed and showed a high PDI value that may be attributed to the presence of some of the Eug-NE droplets in aggregates. Eug-NE particles were nearly spherical when examined by transmission electron microscopy, with a diameter ranging from 20 to 80 nm. The absence of intermolecular interactions among all ingredients was confirmed using Fourier-transform infrared spectroscopy.

### 2.3. Preparation of ABZ and Eug Formulations for the In Vivo Study

Each formulation was freshly prepared before administration. ABZ pure standard was dissolved in phosphate-buffered saline PBS (pH 7.4) by vortex producing an ABZ suspension (2.5 mg/mL) [6]. Eug essential oil and nanoemulsion were dissolved in 0.1% DMSO and the stock solutions were prepared to reach a final concentration of 100 μg/mL. The mixture was adequately homogenized by a magnetic stirrer.

### 2.4. Parasite Material and Protoscolex Collection

Protoscoleces were aseptically collected from the liver and lung hydatid cysts isolated from infected camels slaughtered in Bani-Adi and El-Atamina abattoirs, Assiut Governorate, Egypt, as described previously [31]. The viability of PSCs was tested before inoculation by the eosin exclusion test. Samples with >95% viability were used in the experiment.

### 2.5. Experimental Design

Forty-eight specific pathogen-free male Wistar rats (12–16 weeks old) weighing 180–200 g, purchased from the animal house of the Faculty of Medicine, Assiut University, were used in the experiment. Rats were housed under standard laboratory conditions. Rats were randomly assigned into eight groups (six rats per group). A murine model of cystic echinococcosis was established by an intraperitoneal (IP) injection of 3000 viable PSCs resuspended in 500 μL of sterile HBSS [41,42]. Four months after infection, the infected rats were divided into treated and control groups as follows.

(1) CE control group [received PBS];
(2) Eug/CE group [infected rats treated daily with an intragastric dose of 100 μL of 10% Eug oil (500 μg/kg/day) [25]];
(3) Eug-NE/CE treated group [infected rats treated daily with an intragastric dose of 100 μL of 10% Eug-NE (500 μg/kg/day)];
(4) ABZ/CE group [infected rats treated with an intragastric administration of 500 μL (10 mg/kg/day) of ABZ suspension [42]].

**Figure 1:**
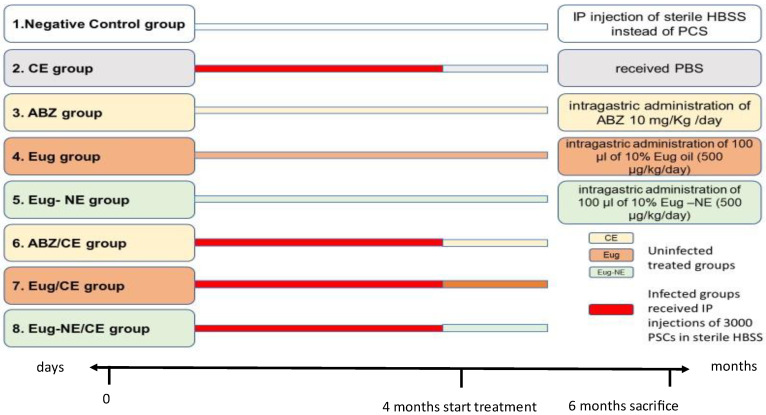
Experimental study plan.

All treatments and the control were given to rats by intragastric intubation. In addition, four uninfected groups included the negative control (received IP injection of sterile HBSS instead of PSCs), ABZ, Eug, and Eug-NE uninfected treated groups that received the same drug regimens as infected treated groups and for the same duration. The experimental study plan is shown in Figure 1.

Two months after treatment, all rats were sacrificed, a necropsy was carried out immediately, and rats were examined for the following tests.

### 2.6. Assessment of Cyst Development

The peritoneal cavity was opened carefully and examined for hydatid cysts. The liver, lungs, brain, and spleen were isolated from each rat, examined macroscopically for CE, and weighed. The viability of the protoscoleces in the isolated cysts was assessed. The organ hypertrophy indicator for these organs was calculated according to the following formula [43]:

$$\mathrm{Organ}\;\mathrm{hypertrophy}\;\mathrm{indicator}\;=\frac{\mathrm{Organ}\;\mathrm{weight}}{\mathrm{Animal}\;\mathrm{weight}-\;\mathrm{Hydatid}\;\mathrm{cyst}\;\mathrm{weight}}\times 100$$

### 2.7. Histopathological and Immunohistochemical (IHC) Evaluation

#### 2.7.1. Histopathological Analysis

Liver and lung specimens were collected and fixed in 10% neutral buffered formalin, mounted in paraffin blocks, and cut into 5 μm thick sections. The sections were stained with Mayer’s hematoxylin (Merck, Darmstadt, Germany) and eosin (Sigma, St. Louis, MO, USA), and studied microscopically to examine the presence of CE and evaluate the structural alterations of hepatic parenchymal cells and lung tissue. Additionally, histochemical staining with Sirius red stain was performed to evaluate the collagen fiber content [44] according to the manufacturer’s protocol.

Histopathological examination to assess hepatic changes (hepatocyte necrosis, hepatocyte vacuolation, and lymphoid cell infiltration) and lung changes (alveolar wall thickness, lymphoid cell infiltration, and congestion) was performed blindly on coded samples compared to the control group. Histopathological scoring was performed based on a 0-to-3 scale, as follows: 0 = normal; 1 = (<25% damage) focal, slight changes; 2 = (25–50% damage) multifocal, significant changes; and 3 = (>50% damage) common widespread changes. The percentage of positive collagen area was calculated with ImageJ using threshold area fraction determination. The amount of collagen was reported from the total number of pixels in the optical view as a percentage and expressed as mean ± standard deviation [45,46].

#### 2.7.2. IHC Analysis

IHC analysis of the transcription markers GATA3 and STAT4 was performed on formalin-fixed, paraffin-embedded samples using the UltraVision LP Large Volume Detection System (Thermo Fisher Scientific, Santa Clara, CA, USA). The slides were incubated with rabbit anti-STAT4 and GATA3 polyclonal antibodies at a 1:100 dilution. Indirect immunoperoxidase staining was performed according to the manufacturer’s instructions (Invitrogen, Thermo Fisher Scientific, Santa Clara, CA, USA). The sections were counterstained with Mayer’s hematoxylin, and the slides were observed under a microscope to check for positive reactions.

STAT4 and GATA3 immunostaining was scored on a semiquantitative scale by evaluating the intensity and percentage of positively stained cells on 10 high-power fields. The intensity of STAT4 and GATA3 staining was scored as follows: 0 = none, 1 = weak, 2 = moderate, and 3 = strong. The percentage of positively stained cells was assigned according to the following: 0 = 0%, 1 = 10%, 2 = 10–30%, and 3 = >30%.

### 2.8. Plasma Cytokine Measurement

At the end of the experiment, blood samples were collected from each rat, and sera were isolated by centrifugation at 1500× *g* for 15 min and preserved at −20 °C for further analysis. The IL-4 and IFN-γ serum levels in the experimental animals were calculated against standard curves using commercial ELISA kits (OriGene^®^ Technologies, Inc., Rockville, MD, USA) according to the manufacturer’s instructions. Optical density was read at a wavelength of 450 nm using a microplate reader (Thermo Fisher Scientific, Santa Clara, CA, USA), and the concentration was calculated.

### 2.9. Statistical Analysis

Statistical analyses were performed using IBM-SPSS 24.0 (IBM-SPSS, Inc., Chicago, IL, USA). Results were reported as the mean ± standard deviation and percentage. To evaluate the mean differences among the tested groups, one-way analysis of variance (ANOVA) and repeated-measures ANOVA were performed. A posthoc test was performed using Bonferroni corrections for comparison among the studied groups. Spearman’s rank correlation test was performed to test the correlation among the tested variables. *p* ≤ 0.05 was considered significant.

## 3. Results

### 3.1. Assessment of Hydatid Cyst Development

**Figure 2:**
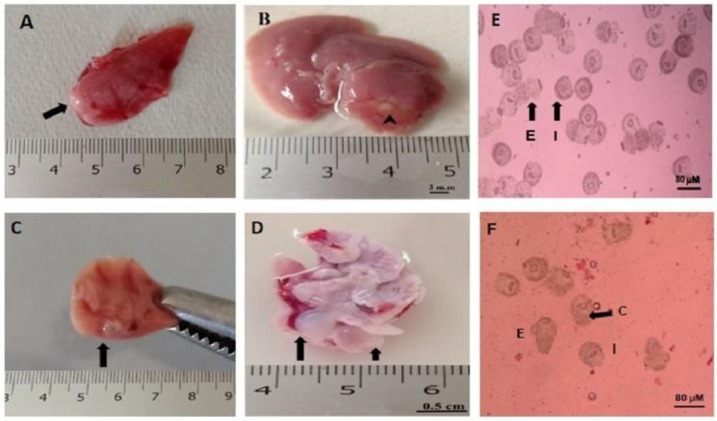
Secondary hydatid cysts in internal organs of infected rats. (**A**,**B**) Liver with hydatid cysts (arrowhead). (**C**,**D**) Lungs with hydatid cysts (arrows). (**E**,**F**) Aspirate of rat liver cyst of the infected nontreated group stained with 0.1% eosin (**F**). Dead protoscoleces stained red (protoscolex viability was 100%; I, invaginated viable protoscoleces, E, evaginated protoscoleces; C, calcareous corpuscles).

A CE murine model was conducted in rats. At the end of the experiment, all rats were euthanized. At necropsy, the peritoneal cavity of infected control rats (CE group) was inspected, and the organs (liver, lung, brain, and spleen) were removed and examined for secondary hydatid cysts. The liver and lungs showed multiple macroscopic cysts, whereas the brain and spleen showed no gross cysts. Protoscoleces aspirated from secondary hydatid cysts were 100% viable, as confirmed by the eosin exclusion test (Figure 2).

**Figure 3:**
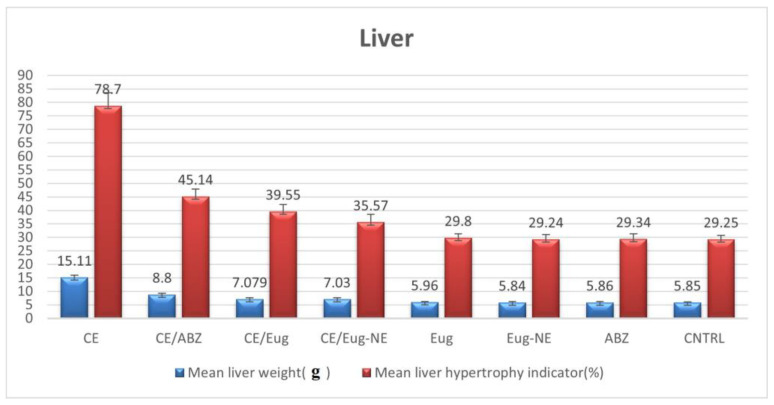
Mean liver weight (g) and liver hypertrophy indicator (%).

**Figure 4:**
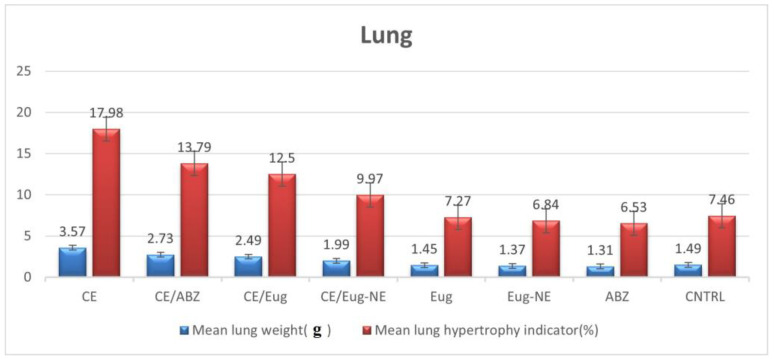
Mean lung weight (g) and lung hypertrophy indicator (%).

Table 1 shows the difference between the cyst weights (mean ± SD) recorded after treatments on the different experimental groups (the control CE group and infected treated groups) in the liver and lungs. The results showed a statistically significant reduction in cyst weights in all treated groups, especially in the Eug-NE/CE treated group followed by the Eug/CE treated group in lung and liver tissues Additionally, the efficacy of treatment was evaluated by measuring the organ hypertrophy indicator, which revealed a significant reduction in organ weights and hypertrophy indicator for liver and lung tissue in all treated groups compared to that of the infected untreated group (*p* < 0.01). Eug-NE was the most effective in reducing the hypertrophy indicator, followed by Eug and ABZ, as shown in Figure 3 and Figure 4 and “Tables S1–S4”.

### 3.2. Histopathological Analysis

#### 3.2.1. Histopathological Changes in the Liver

**Figure 5:**
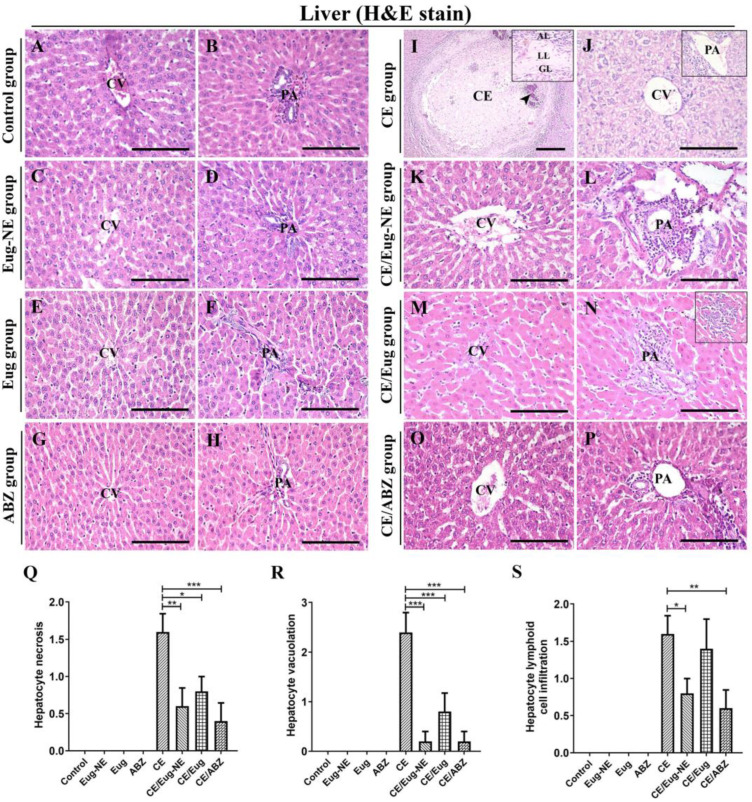
Photomicrographs of the histopathological lesions in the liver. (**A**–**H**) Liver sections from the negative control group (**A**,**B**) and the positive control groups Eug-NE (**C**,**D**), Eug (**E**,**F**), and ABZ (**G**,**H**) show the normal histological structure of hepatic cells arranged in cords separated by hepatic sinusoids and radiating from the central vein (CV) to the portal area (PA) (containing branches of hepatic artery, portal vein, and bile duct). (**I**,**J**) Liver sections from the CE group show hydatid cysts (cystic echinococcosis, CE) with calcification (arrowhead) (**I**). The cyst comprises the germinal layer (GL), laminated layer (LL), and adventitial layer (AL) (inset). (**J**) Liver cells showing vacuolation and necrosis, and lymphoid cell infiltration in the portal area (inset). (**K**–**P**) Liver sections from CE/Eug-NE, CE/Eug, and CE/ABZ groups showed necrosed hepatocytes in some areas (**K**,**M**,**O**) and lymphoid cell infiltration in the portal area (PA) (**L**,**N**,**P**). Focal lymphoid cell infiltration in the hepatic parenchyma in CE/Eug group (**N**) (inset). (**Q**–**S**) Evaluation of hepatic lesions based on hepatocyte necrosis, vacuolation, and lymphoid cell infiltration (*p* < 0.05). Hematoxylin and eosin stain. Bars (**A**–**H**) and (**J**–**P**) = 50 µM. Bar (**I**) = 200 µM. * *p <* 0.05, ** *p <* 0.001, *** *p <* 0.0001.

Histological examination of liver sections from the negative control group (Figure 5A,B) or other control groups (treated with Eug-NE, Eug, or ABZ; Figure 5C–H) demonstrated the normal hepatic structure of lobules. In the CE group, multiple liver sections showed variable-sized hydatid cysts (CE). Cysts were located at the edge of or inside the liver. Cysts were composed of a thin inner lining germinal layer (GL), a cellular eosinophilic laminated layer consisting of a polysaccharide–protein complex, and an outer adventitial layer consisting of fibrovascular tissue with a variable number of inflammatory cells (Figure 5I). Adjacent liver cells often showed pressure atrophy changes. In most cysts, the hydatid material was no longer visible, and the center consisted of necrotic cellular debris that was variably mineralized (Figure 5I). Furthermore, other severe histopathological abnormalities were observed in the liver sections of the CE group, including vacuolation of the hepatocyte cytoplasm, hydropic degeneration, and necrosis with karyolytic nuclei (Figure 5J). In addition, there was severe infiltration of lymphoid cells in the portal area (Figure 5J, inset) and multiple focal lymphoid cell aggregations in the parenchyma.

However, Eug-NE, Eug, or ABZ administration to rats with CE improved the histological structure in the liver sections (Figure 5K–S). These observations correlated with a substantial decrease in hydatid cysts (Figure 5K–P). Histopathological evidence of hepatocyte cytoplasmic vacuolation and hepatocyte degeneration and necrosis markedly regressed, with only a few sporadic hepatocytes displaying necrosis (Figure 5K,M,O). Infiltration of lymphoid cells was mild to moderately confined to the portal area (Figure 5L,N,P). However, mild focal areas of lymphoid cell aggregation were seen in the hepatic parenchyma of the CE/Eug group (Figure 5N, inset). Histopathological scoring of hepatic lesions, including hepatocyte necrosis, hepatocyte vacuolation, and lymphoid cell infiltration, revealed significantly fewer hepatic lesions in the infected treated groups than in the untreated group (CE group). CE/ABZ and CE/Eug-NE groups showed lower lesions than the CE/Eug group, but the difference between the three groups was insignificant (Figure 5Q–S).

#### 3.2.2. Histopathological Changes in the Lungs

**Figure 6:**
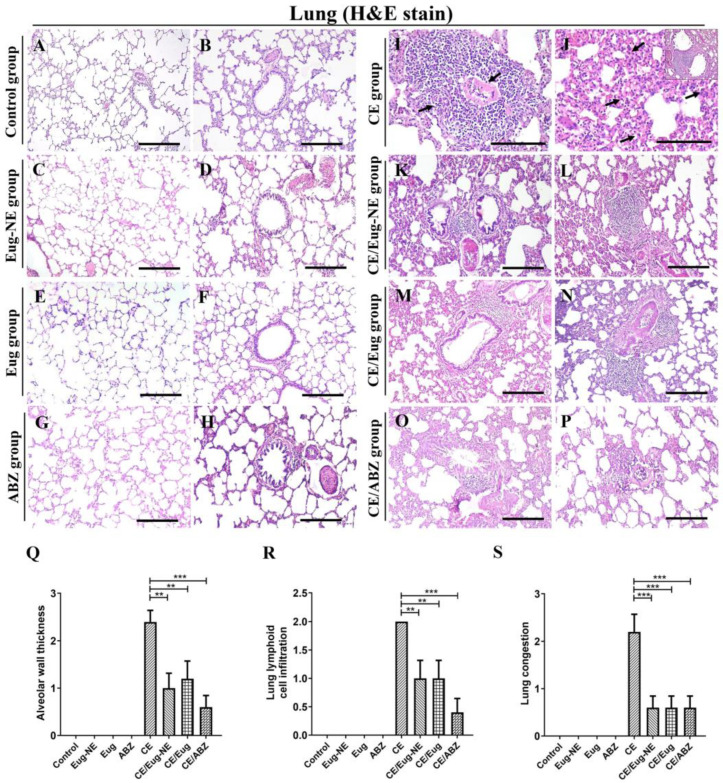
Photomicrographs of the histopathological lesions in the lung. (**A**–**H**) Lung sections from the negative control group (**A**,**B**) and the positive control groups Eug-NE (**C**,**D**), Eug (**E**,**F**), and ABZ (**G**,**H**) the normal histological structure of the lung parenchyma, consisting of thin-walled alveoli and bronchiole. (**I**,**J**) Lung sections from CE group show degenerating scolices (arrows) with lymphoid cell infiltration (**I**). (**J**) The thickness of the alveolar wall (arrows), and lymphoid cell infiltration around the bronchioles (inset). (**K**–**P**) Lung sections from CE/Eug-NE, CE/Eug, and CE/ABZ groups showed mild thickening of the alveolar wall (**K**,**M**,**O**), and lymphoid cell infiltration around the bronchioles (**K**,**M**,**O**) and blood vessels (**L**,**N**,**P**). (**Q**–**S**) Evaluation of lung lesions based on alveolar wall thickness, lymphoid cell infiltration, and congestion (*p* < 0.05). Hematoxylin and eosin stain. Bars (**A**–**H**,**K**–**P**) = 100 µM. Bars (**I**,**J**) = 50 µM. ** *p* < 0.001, *** *p* < 0.0001.

Histological examination of lung sections from the negative control group (Figure 6A,B) or other control groups (treated with Eug-NE, Eug, or ABZ; Figure 6C–H) showed the normal structure of lung parenchyma, consisting of thin-walled alveoli and cuboidal epithelium-lined bronchioles.

In the CE group, multiple lung sections showed degenerating scoleces surrounded by a thick layer of lymphoid cells (Figure 6I). The lung parenchyma adjacent to the cyst was atelectatic. The associated changes in the lungs were chronic interstitial pneumonia characterized by severe diffuse thickening of the alveolar septa (Figure 6J). Additional findings included severe aggregation of lymphoid cells in the peribronchiolar (Figure 6J, inset) and perivascular areas, edema appeared as eosinophilic fluid, congestion, intimal and medial thickening in the blood vessels, and alveolar emphysema and atelectasis.

However, the severity of histopathological lesions in the lungs from the CE/Eug-NE, CE/Eug, and CE/ABZ groups was reduced with the absence of degenerating scoleces, compared to that of the CE group (Figure 6K–P,Q–S) and showed a mild thickness of the alveolar septa and mild-to-moderate aggregation of lymphoid cells in the peribronchiolar (Figure 6K,M,O) and perivascular areas (Figure 6L,N,P).

Histopathological scoring of lung lesions showed that alveolar wall thickness and lymphoid cell infiltration significantly decreased in the infected treated groups compared to that of the infected untreated group (CE group). The CE/ABZ group showed insignificantly lower lesions than those of the CE/Eug-NE and CE/Eug groups, with no difference in congestion between these groups (Figure 6Q–S).

### 3.3. Histochemical Evaluation of Collagen Fiber Deposition in the Liver

**Figure 7:**
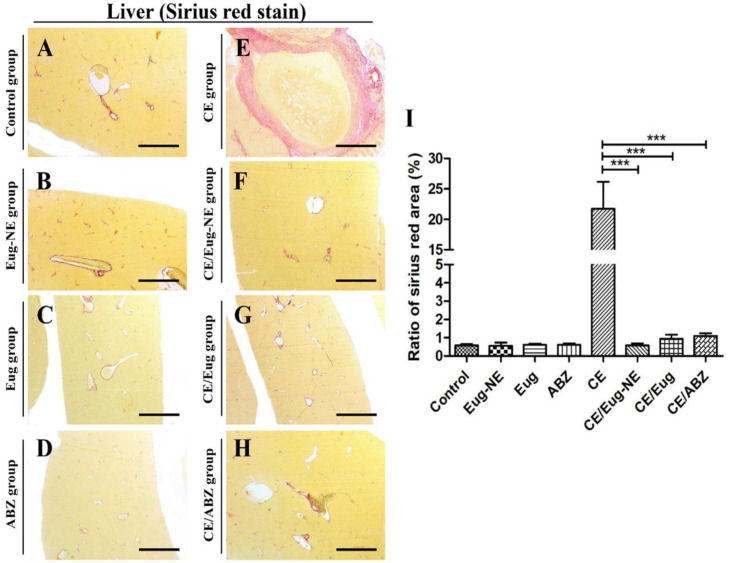
Photomicrographs of histochemical evaluation of collagen deposition and fibrosis in the liver by Sirius red stain. (**A**–**H**) Staining of the collagen fibers with Sirius red stain in liver sections from different groups with red color. Sirius red stain. Bars = 500 µM. (**I**) The fibrotic area was quantified by determining the ratio of Sirius red area to total area (%) using ImageJ. *** *p <* 0.0001.

CE is known for its fibrocystic reaction in the infected liver because of the host immune response that considers the cyst a foreign body. This study further examined collagen fiber formation by staining the liver sections with Sirius red. There was an absence of collagen deposition in the liver sections from the control groups (negative, Eug-NE, Eug, and ABZ; Figure 7A–D). However, the CE group showed severe deposition of collagen fibers that was mainly localized around the cysts (Figure 7E). Hepatic parenchyma immediately adjacent to the cystic structures was replaced by fibrosis and infiltrated by inflammatory cells (Figure 7E). The extent of collagen fiber deposition (Figure 7F–H) and the percentage of fibrosis significantly decreased upon Eug-NE, Eug, or ABZ administration (Figure 7I). The fibrosis in the CE/Eug-NE group was lesser than in the CE/Eug and CE/ABZ groups, but the difference was statistically insignificant (Figure 7I).

### 3.4. IHC Analysis

**Figure 8:**
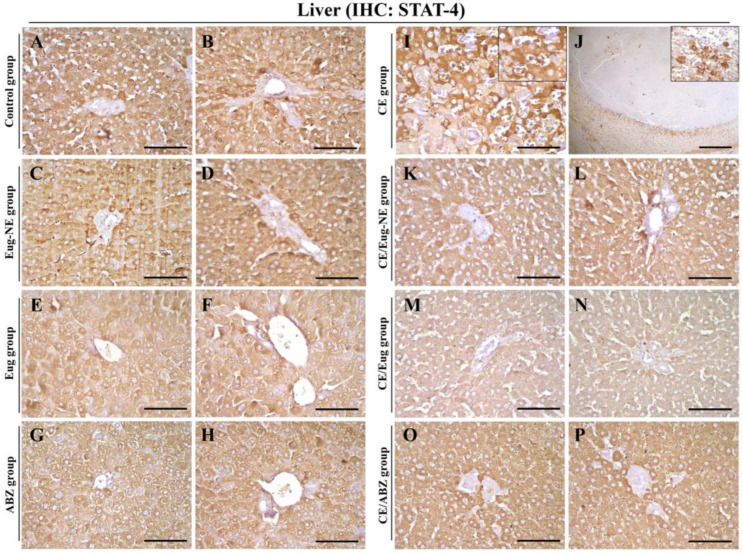
Photomicrographs of STAT4 immunostaining in the liver. (**A**–**P**) Liver sections from different groups showed negative STAT-4 immunostaining, except the CE group showing STAT-4 immunostaining in the hepatocytes (**I**) (inset) and lymphoid cells around the hydatid cysts (**J**) (inset). Bar (**J**) = 200 µM. Bars (**A**–**I**,**K**–**P**) = 50 µM.

**Figure 9:**
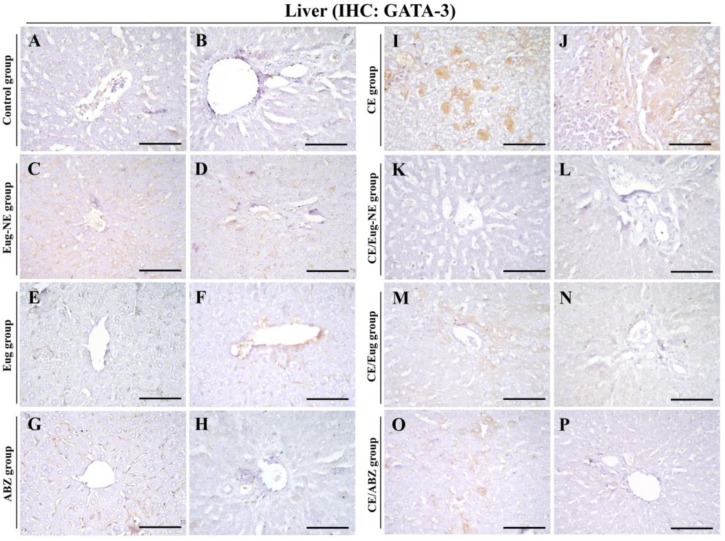
Photomicrographs of GATA3 immunostaining in the liver. (**A**–**P**) Liver sections from different groups showed negative GATA-3 immunostaining, except the CE group showed GATA-3 immunostaining in the hepatocytes (**I**) and lymphoid cells around the hydatid cysts (**J**). Bars (**A**–**P**) = 50 µM.

IHC analysis was performed in the liver sections using STAT4 and GATA3 antibodies. STAT4 (Figure 8I,J) and GATA3 (Figure 9I,J) were positive in the liver and inflammatory cells surrounding the cysts in the CE group compared to those in the negative control group (Figure 8A,B and Figure 9A,B) and treated uninfected control groups (Eug-NE, Eug, and ABZ; Figure 8C–H and Figure 9C–H). This increased expression in liver sections of the CE group was attenuated by treatment of Eug-NE, Eug, or ABZ (Figure 8K–P and Figure 9K–P). IHC results were further evaluated according to the intensity and number of positive cells. Compared to that in the CE group, the intensity of STAT4 and the number of STAT4- and GATA3-positive cells were significantly decreased in the CE/Eug-NE, CE/Eug, and CE/ABZ groups, whereas the intensity of GATA3 was significantly decreased only in the CE/Eug-NE group (Table 2).

### 3.5. Plasma Cytokine Measurement

To assess the host immune response after treatment, serum cytokine levels of IFN-γ and IL-4 were measured, reflecting Th1 and Th2 cytokine profiles, using ELISA in the infected untreated (CE) group (638.93 ± 13.7) compared to the infected treated groups. In this study, all treatment regimens had significantly increased IFN-γ levels in the treated uninfected groups with Eug, Eug-NE, or ABZ (209.02 ± 9.7, 361.53 ± 14.4, and 251.50 ± 11.9, respectively). However, the infected treated groups had a nearly threefold increase in serum IFN-γ levels compared to those of the treated uninfected controls. Serum IFN-γ levels were significantly (*p* < 0.001) higher in infected rats treated with ABZ (881.9 ± 19.2 pg/mL), followed by Eug-NE (794.1 ± 17 pg/mL) and Eug (764.9 ± 17.5 pg/mL; Table 3).

In addition, serum IL-4 concentration significantly decreased (*p* = 0.003) in the Eug-NE treated group (11.8 ± 1 pg/mL) compared to that in the infected untreated (CE) group, whereas groups treated with ABZ and Eug showed a statistically insignificant decrease (13.95 ± 1.2 and 13.58 ± 1.3 pg/mL, respectively; Table 4).

## 4. Discussion

ABZ is the principal anthelminthic drug used for CE treatment. ABZ has recently shown many adverse effects, with some limitations and low efficacy [43,47,48]. Therefore, novel and complementary scolicidal agents are urgently needed [13].

Following an initiative by the World Health Organization in August 2000, the opportunity to evaluate many medicinal plants and natural products in validated antiparasitic screens will help develop new pharmaceuticals with high safety and efficacy [49]. Plant essential oils can be alternatives or adjuncts to current antiparasitic therapies [50].

Based on the promising scolicidal effects of Eug and its nanoemulsion against protoscoleces of hydatid cysts observed in a previous study [31], this study investigated the antihydatic properties of both preparations in a murine model.

This study revealed the development of secondary hydatid cysts, mainly in the liver and lungs of infected untreated (CE) rats, with 100% viable protoscoleces on aspiration. This was in agreement with many previous studies that reported that the liver (70%) and lungs (20%) are the primary sites of CE involvement [51,52].

However, oral administration of 500 μg/kg of 10% Eug-NE or Eug once daily for three months significantly inhibited hydatid cyst development in all treated rats. They also significantly decreased all cyst weights, organ weights, and hypertrophy indicators in the infected and treated groups, with Eug-NE being more effective, followed by Eug and ABZ (*p* < 0.01). Our results were consistent with previous studies that demonstrated that Eug oil is an effective antiparasitic in vivo. Eug was previously tested in the treatment of *Leishmania donovani*-infected mice and showed an effective reduction of the parasite load in hepatic and splenic tissue, with an enhanced Th1 immune response [53,54]. It also suppressed *Trypanosoma cruzi* parasitemia in mice with a higher cure rate than benznidazole, the reference drug [55]. Furthermore, Eug disrupted the erythrocytic cycle of *Plasmodium falciparum*, reducing the risk of cerebral malaria [56]. As an anthelminthic, Eug was investigated in a murine model of *Schistosoma mansoni* and revealed a significant reduction in the overall worm burden by 19.2%, improving inflammatory and fibrotic responses [25].

To the authors’ knowledge, the anti-inflammatory effects of Eug-NE have been underinvestigated in vivo. A gel preparation of Eug-NE was studied as an anti-inflammatory, analgesic, and anesthetic in treating induced gingivitis in rats and showed significant results by reducing tumor necrosis factor-α (TNF-α) and IL-1β [57]. It also significantly improved carrageenan-induced paw edema in rats compared to the commercially available piroxicam gel [58].

Several plant extracts have been tested in vivo against CE and revealed high efficacy; however, only a few essential oils were used. The commonly tested essential oils against CE in vivo include carvacrol [59], β-myrcene [6] essential oils, *Cuminum cyminum* seeds [60], and *Zataria multiflora* [13,61], *Zingiber officinale* [43], *Allium sativum* [62], *Punica granatum* peel [10], and *Ziziphora tenuior* [63] plant extracts.

The application of a nanopreparation against CE in vivo has also been limited. Moazeni et al. [14] tested the therapeutic effects of *Z. multiflora* nanoemulsion in vivo and revealed a reduction in hydatid cyst burden. In another study, cerium oxide nanoparticles showed a decreased mean weight and size of hydatid cysts in treated mice [64].

Histopathologically, liver sections from the CE group showed variable-sized hydatid cysts with pressure atrophy of adjacent liver cells. Hepatic cells adjacent to the cysts were replaced by fibrosis and infiltrated by inflammatory cells, resulting in the destruction of the hepatic parenchyma, as reported previously [42,62,65]. The infiltration of inflammatory cells, particularly macrophages, plays an important role in the initiation and formation of fibrosis [66], whereas the severe destruction of the hepatic architecture is attributed to the extensive infiltration of inflammatory cells and the formation of fibrosis caused by the CE-induced immune imbalance in hepatic tissue [67].

However, a substantial decrease in hydatid cysts and a significant regression (*p* < 0.05) of histopathological changes and inflammatory reactions in liver sections were observed in infected groups treated with ABZ, followed by Eug-NE and Eug, but the difference among the three drugs was insignificant. This was probably related to the antioxidant and anti-inflammatory effects of Eug reported in many studies. A *S. mansoni* murine model treated with Eug showed decreased number and size of liver granulomas and a reduced chronic inflammatory cell infiltrate [25]. Treatment of experimental visceral leishmaniasis with Eug oleate resulted in the absence of hepatic granulomas, indicating the clearance of infection inside the hepatic tissue [54]. Fathy et al. [68] and Yogalakshmi et al. [69] stated that treated rats with Eug after induced liver injury showed reduced fatty changes, hepatic degeneration, hepatic fibrosis, and lipid peroxidation indices. Additionally, some inflammatory markers were reduced, including cyclo-oxygenase-2, TNF-α, and IL-6 expression, and there was an improvement in the antioxidant status. Eug also reduced in vitro and in vivo leukocyte migration by modulating adherence to perivascular tissue, without affecting cell viability [70].

These observations correlated with a less fibrotic reaction in infected groups treated with Eug-NE, followed by Eug and ABZ. Eug is known for its antifibrotic effect, as reported in many studies. This agreed with El-kady et al. [25], who reported a significant reduction in collagen fiber deposition in a murine model of *S. mansoni* treated with Eug. Moreover, mice with induced liver cirrhosis treated with Eug showed a significant decrease in liver enzymes, with considerable improvement in the hepatic tissue architecture [71]. The mechanism of the antifibrotic effects of Eug was attributed to its ability to inhibit nitric oxide synthesis and fibrogenic cytokine expression, reducing hepatic inflammation and fibrosis, indicating that Eug could be a potential therapeutic agent for some liver diseases with intervening fibrosis [68].

In addition, lung sections in the CE group showed degenerating scoleces surrounded by a thick layer of lymphoid cells. The lung parenchyma adjacent to the cyst was atelectatic. Chronic interstitial pneumonia associated with severe aggregation of lymphoid cells in the peribronchiolar and perivascular areas, edema, congestion, intimal and medial thickening in blood vessels, and alveolar emphysema and atelectasis were additional findings. This agreed with Beigh et al. [72] and Ramos et al. [73], who reported similar lung changes in CE-infected lungs.

However, the severity of histopathological lesions of the lungs from the treated groups was reduced by the absence of degenerating scoleces. Alveolar wall thickness and lymphoid cell infiltration were significantly (*p* < 0.05) decreased in the CE/ABZ, CE/Eug-NE, and CE/Eug groups, with no difference in congestion between these groups. Magalhães et al. [74] reported that Eug treatment of lipopolysaccharide-induced lung injury causes a reduction in alveolar collapse, neutrophil infiltration, and collagen fiber deposition in the lung parenchyma. This may be attributed to the antioxidant and anti-inflammatory effects of Eug produced in the lung tissue by the downregulation of proinflammatory cytokine profiles such as IL-6, IL-1β, and TNF-α expression and nuclear factor-κB and activator protein-1 signaling pathways [75].

Indeed, the evaluation of the treatment response to a chemotherapeutic agent in CE depends on different parameters, including an assessment of the host immune response. The immune response to CE infections, as well as other helminthic infections, involved Th1 and Th2 cell populations substantially expressed in hydatid infections [67]. Th1 cells produce several cytokines such as IL-2, IFN-γ, and lymphotoxin, whereas Th2 cells express other markers such as IL-4, IL-5, IL-6, and IL-10. They have a cross-inhibitory mode of action. IFN-γ suppresses Th2 cell proliferation, whereas IL-10 inhibits Th1 cytokine production [76]. This was consistent with the results of the serum IFN-γ and IL-4 levels, which reflected Th1 and Th2 immune responses. Both cytokines significantly increased (*p* < 0.001) in the infected untreated group (CE group) compared to those in the control negative group. This was confirmed by IHC analysis in the liver sections using STAT4 and GATA3 markers, representing Th1 and Th2, respectively. STAT4 and GATA3 were expressed in the liver and inflammatory cells surrounding hydatid cysts in the CE group compared to the negative control group. Surprisingly, hydatid infection induced high Th1 and Th2 cytokine levels, although the two subsets inhibited each other. This might be related to the complexity of different hydatid fluid antigens, which contain different epitopes for each T-cell subset [67].

Furthermore, the immune system response to CE varies according to the clinical features of the disease and response to treatment. Riganò et al. [36] analyzed the cytokine profile of several patterns of T-cell populations derived from patients with CE at different clinical stages of hydatid disease. They observed that the T-cell population derived from patients with inactive hydatid cysts predominantly produced IFN-γ; however, patients with active or transitional cysts showed exclusive Th2 cytokine expression. Additionally, infected BALB/c mice with dead hydatid cysts showed elevated IFN-γ levels, suggesting their role in destroying protoscoleces and established cysts [77]. In human subjects treated with ABZ, a Th1 cytokine profile was dominant [78]. However, Bayraktar et al. [79] reported that IL-4 significantly increased in patients with CE and active cysts compared to healthy controls and decreased with treatment.

In the same context, Shaheen et al. [80] investigated the T-cell pattern of CE at the tissue level by IHC in Egyptian patients resistant to different treatments to identify the dominant T-cell population. They found a higher local expression of the GATA3 transcription marker (expressed by Th2 cells) in relation to the STAT4 marker (expressed by Th1 cells) in all studied groups, reflecting the significant dominance of the Th2 subset on the Th1 response in cases with treatment failure.

This study was consistent with previous studies, as serum IFN-γ levels significantly increased (*p* < 0.001) in infected rats in response to treatment with ABZ, followed by Eug-NE and Eug. IFN-γ elevation was reported after Eug treatment of a murine model with experimental visceral leishmaniasis in several studies [40,54,81]. This was confirmed by estimating IFN-γ levels in the uninfected groups treated with Eug and Eug-NE compared to the control negative group. Results showed that IFN-γ levels were significantly elevated. A marked reduction in hydatid cyst weight was observed in all infected treated groups, wherein IFN-γ levels were raised. The protective role of IFN-γ was attributed to the fact that IFN-γ activates peritoneal macrophages and local antibodies that can recognize protoscolex antigens and activate the complement system, resulting in the elimination of ~90% of parasite inoculum [82]. At the tissue level, STAT4 expression in liver sections of the CE group was attenuated by treatment with Eug-NE, Eug, or ABZ. However, IFN-γ levels increased in these groups. This could be explained by a previous observation indicating greater activation of NK cells in the peripheral blood of patients with active CE than in controls with increased IFN-γ production [83]. Moreover, Vishteh et al. [84] reported that Eug enhanced NK cell activity in mice.

Additionally, serum IL-4 concentration significantly decreased in the infected group treated with Eug-NE compared to that in the CE group but insignificantly decreased in groups treated with ABZ and Eug. GATA3 expression in CE liver sections was also reduced by treatment with Eug-NE, Eug, or ABZ, confirming the decrease in serum IL-4 levels in these groups. This agreed with Islamuddin et al. [40], who reported that Eug administration decreased IL-4 levels in mice with experimental visceral leishmaniasis. Mice with CE treated with alkaloids and ABZ showed decreased IL-4 levels compared to the untreated infected control group [85].

## 5. Limitations of the Study

In this study, there is a lack of information on the toxicology and pharmacology of Eug oil and its nanoemulsion and their effects on hepatic enzymes and/or kidney function tests. Additionally, an assessment of the stability of the nanoformulation was not performed. A study of the effect of combining the tested formulations with the standard drug (ABZ) was lacking. Further study concerning the uses of different treatment regimens and durations is highly recommended to confirm the effects of Eug oil and nanoemulsion. In addition, future studies are encouraged to evaluate the effect of these preparations on different inflammatory cytokines and cellular transcription factors to better understand the exact mechanism of Eug oil and Eug-NE in the treatment of cystic echinococcosis and explore their immunomodulatory effects.

## 6. Conclusions

To the authors’ knowledge, Eug and Eug-NE were used for the first time against CE in vivo and showed a better improvement in the histopathological structure and fibrosis, with a significant reduction in cyst weight and organ hypertrophy upon treatment with Eug-NE, Eug, and ABZ. Moreover, Eug and Eug-NE improved the immune response at systematic and tissue levels, as they significantly increased IFN-γ levels, which are protective against CE, and decreased IL-4 levels, reflecting a good treatment response. IHC analysis demonstrated a significant reduction in STAT4 and GATA3 expression in all treated groups, suggesting their role in modulating the immune response. Generally, Eug-NE revealed high effectiveness in preventing the progression of hydatid cysts in vivo, with a significant reduction in liver fibrosis, compared to that of Eug and ABZ. Eug showed a similar in vivo effect to Eug-NE but to a lesser extent. Consequently, these formulations could be alternative or complementary antihydatic agents in hydatid cyst treatment.

## Figures and Tables

**Table 1:** Comparison of the mean (±SD) weight of hydatid cysts recovered from the liver and lung of CE infected rats from the treated and control groups.

Experimental Groups	Cyst Weight (g) Mean ± SD	
	Liver	Lung
CE control group	9.260 ± 0.928	2.077 ± 0.269
ABZ/CE treated group	2.952 ± 0.526 **	1.239 ± 0.216 **
Eug/CE treated group	1.941 ± 0.505 **	1.007 ± 0.229 **
Eug-NE/CE treated group	1.187 ± 0.570 **	0.498 ± 0.148 **

** One-way ANOVA test. *p* value < 0.001 was statistically significant differences between the treated group vs. the control group.

**Table 2:** IHC score of STAT4- and GATA3-positive cells in liver tissues.

Experimental Groups	Immunohistochemistry Score (Mean ± SE)			
	STAT-4		GATA-3	
	Intensity	Positive Cells	Intensity	Positive Cells
1. Noninfected control	0.00 ± 0.00 ^(b)^	0.00 ± 0.00 ^(b)^	0.00 ± 0.00 ^(b)^	0.00 ± 0.00 ^(b,d)^
2. Infected control (CE)	2.33 ± 0.33 ^(a,c,d,e,f,g,h)^	2.67 ± 0.33 ^(a,c,d,e,f,g,h)^	1.67 ± 0.33 ^(a,c,f,g,h)^	2.00 ± 0.00 ^(a,c,d,e,f,g,h)^
3. Infected treated (CE/Eug-NE)	0.67 ± 0.33 ^(b)^	0.67 ± 0.33 ^(b)^	0.33 ± 0.33 ^(b)^	0.67 ± 0.33 ^(b)^
4. Infected treated (CE/Eug)	0.67 ± 0.33 ^(b)^	0.67 ± 0.33 ^(b)^	0.67 ± 0.33	1.00 ± 0.00 ^(a,b,f,g,h)^
5. Infected treated (CE/ABZ)	0.67 ± 0.33 ^(b)^	0.67 ± 0.33 ^(b)^	0.67 ± 0.33	0.67 ± 0.33 ^(b)^
6. Uninfected treated (Eug-NE)	0.00 ± 0.00 ^(b)^	0.00 ± 0.00 ^(b)^	0.00 ± 0.00 ^(b)^	0.00 ± 0.00 ^(b,d)^
7. Uninfected treated (Eug)	0.00 ± 0.00 ^(b)^	0.00 ± 0.00 ^(b)^	0.00 ± 0.00 ^(b)^	0.00 ± 0.00 ^(b,d)^
8. Uninfected treated (ABZ)	0.00 ± 0.00 ^(b)^	0.00 ± 0.00 ^(b)^	0.00 ± 0.00 ^(b)^	0.00 ± 0.00 ^(b,d)^

^a^: Significant difference with control group (*p* < 0.05). ^b^: Significant difference with CE group (*p* < 0.05). ^c^: Significant difference with CE/Eug-NE group (*p* < 0.05). ^d^: Significant difference with CE/Eug group (*p* < 0.05). ^e^: Significant difference with CE/ABZ group (*p* < 0.05). ^f^: Significant difference with Eug-NE group (*p* < 0.05). ^g^: Significant difference with Eug group (*p* < 0.05). ^h^: Significant difference with ABZ group (*p* < 0.05).

**Table 3:** Effect of Eug-NE and Eug on the serum IFN-γ levels.

Groups	IFN-γ (pg/mL)	*p*-Value **			
1. Noninfected Control	49.40 ± 4.8	1 vs. 2 < 0.001	2 vs. 4 < 0.001	3 vs. 7 < 0.001	5 vs. 8 < 0.001
2. Infected Control (CE)	638.93 ± 13.7	1 vs. 3 < 0.001	2 vs. 5 < 0.001	3 vs. 8 < 0.001	6 vs. 7 < 0.001
3. Infected treated Eug-NE	794.12 ± 17.0	1 vs. 4 < 0.001	2 vs. 6 < 0.001	4 vs. 5 < 0.001	6 vs. 8 = 0.001
4. Infected treated Eug	764.90 ± 17.5	1 vs. 5 < 0.001	2 vs. 7 < 0.001	4 vs. 6 < 0.001	7 vs. 8 = 0.001
5. Infected treated ABZ.	881.88 ± 19.2	1 vs. 6 < 0.001	2 vs. 8 < 0.001	4 vs. 7 < 0.001	
6. Uninfected treated Eug	209.02 ± 9.7	1 vs. 7 < 0.001	3 vs. 4 = 0.013	4 vs. 8 < 0.001	
7. Uninfected treated Eug-NE	361.53 ± 14.4	1 vs. 8 < 0.001	3 vs. 5 = 0.001	5 vs. 6 < 0.001	
8. Uninfected treated ABZ.	251.50 ± 11.9	2 vs. 3 < 0.001	3 vs. 6 < 0.001	5 vs. 7 < 0.001	
*p*-value *	=0.011				

* *p* < 0.05, one-way ANOVA; ** *p* < 0.05, posthoc test.

**Table 4:** Effect of Eug-NE and Eug on the IL-4 levels.

Groups	IL-4 (pg/mL)	*p*-Value **	
1. Noninfected Control	10.02 ± 0.8	1 vs. 2 < 0.001	3 vs. 5 = 0.037
2. Infected Control (CE)	15.68 ± 1.3	1 vs. 3 = 0.012	3 vs. 8 = 0.003
3. Infected treated Eug-NE	11.82 ± 1.0	1 vs. 4 = 0.007	4 vs. 6 = 0.009
4. Infected treated Eug	13.58 ± 1.3	1 vs. 5 = 0.005	4 vs. 7 = 0.008
5. Infected treated ABZ	13.95 ± 1.2	2 vs. 3 = 0.003	4 vs. 8 = 0.001
6. Noninfected treated Eug	10.67 ± 0.97	2 vs. 6 = 0.001	5 vs. 6 = 0.008
7. Noninfected treated Eug-NE	10.57 ± 0.8	2 vs. 7<0.001	5 vs. 7 = 0.002
8. Noninfected treated ABZ	10.07 ± 0.7	2 vs. 8<0.001	5 vs. 8 = 0.002
		3 vs. 4 = 0.049	
*p*-value *		=0.194	

* *p* < 0.05, one-way ANOVA; ** *p* < 0.05, posthoc test.

## Data Availability

All data are provided in the text without restriction.

## Supplementary Materials

The following supporting information can be downloaded at: https://www.mdpi.com/article/10.3390/tropicalmed8050253/s1, Table S1: Eugenol and eugenol nanoemulsion effects on rat’s liver weight; Table S2: Eugenol and eugenol nanoemulsion effects on rat’s liver hypertrophy indicator; Table S3: Eugenol and eugenol nanoemulsion effects on rat’s lung weight; Table S4.: Eugenol and eugenol nanoemulsion effects on rat’s lung hypertrophy indicator.

## References

[1] Tamarozzi F., Akhan O., Cretu C.M., Vutova K., Fabiani M., Orsten S., Pezzotti P., Popa G.L., Velev V., Siles-Lucas M. (2019). Epidemiological Factors Associated with Human Cystic Echinococcosis: A Semi-Structured Questionnaire from a Large Population-Based Ultrasound Cross-Sectional Study in Eastern Europe and Turkey. Parasites Vectors.

[2] Eckert J., Conraths F.J., Tackmann K. (2000). Echinococcosis: An Emerging or Re-Emerging Zoonosis?. Int. J. Parasitol..

[3] Deplazes P., Rinaldi L., Rojas C.A.A., Torgerson P.R., Harandi M.F., Romig T., Antolova D., Schurer J.M., Lahmar S., Cringoli G. (2017). Global Distribution of Alveolar and Cystic Echinococcosis. Adv. Parasitol..

[4] Agudelo Higuita N.I., Brunetti E., McCloskey C. (2016). Cystic Echinococcosis. J. Clin. Microbiol..

[5] World Health Organization (2010). First WHO Report on Neglected Tropical Diseases: Working to Overcome the Global Impact of Neglected Tropical Diseases.

[6] Fabbri J., Maggiore M.A., Pensel P.E., Albani C.M., Denegri G.M., Elissondo M.C. (2018). Could Beta-Myrcene Be an Alternative to Albendazole for the Treatment of Experimental Cystic Echinococcosis?. Acta Trop..

[7] Karima A., Manel A., Sara B., Assia H., Abdelkarim L., Moussa L., Imene S., Chafia T.B., Ariel N., Katherina V. (2020). International Consensus on Terminology to Be Used in the Field of Echinococcoses. Parasite.

[8] Moro P., Schantz P.M. (2009). Echinococcosis: A Review. Int. J. Infect. Dis..

[9] Pensel P.E., Elissondo N., Gambino G., Gamboa G.U., Benoit J.P., Elissondo M.C. (2017). Experimental Cystic Echinococcosis Therapy: In Vitro and in Vivo Combined 5-Fluorouracil/Albendazole Treatment. Vet. Parasitol..

[10] Labsi M., Khelifi L., Mezioug D., Soufli I., Touil-Boukoffa C. (2016). Antihydatic and Immunomodulatory Effects of Punica Granatum Peel Aqueous Extract in a Murine Model of Echinococcosis. Asian Pac. J. Trop. Med..

[11] Stojkovic M., Zwahlen M., Teggi A., Vutova K., Cretu C.M., Virdone R., Nicolaidou P., Cobanoglu N., Junghanss T. (2009). Treatment Response of Cystic Echinococcosis to Benzimidazoles: A Systematic Review. PLoS Negl. Trop. Dis..

[12] Kang B.S., Choi J.S., Lee S.E., Lee J.K., Kim T.H., Jang W.S., Tunsirikongkon A., Kim J.K., Park J.S. (2017). Enhancing the in Vitro Anticancer Activity of Albendazole Incorporated into Chitosan-Coated PLGA Nanoparticles. Carbohydr. Polym..

[13] Kowsari N., Moazeni M., Mohammadi A. (2021). Effects of Zataria Multiflora Essential Oil on the Germinative Cells of Echinococcus Granulosus. Parasites Vectors.

[14] Moazeni M., Borji H., Saboor M., Jamal M. (2017). In Vitro and In Vivo Antihydatid Activity of a Nanoemulsion of Zataria Multiflora Essential Oil. Res. Vet. Sci..

[15] Moazeni M., Saharkhiz M.J., Hosseini A.A. (2012). In Vitro Lethal Effect of Ajowan (*Trachyspermum ammi* L.) Essential Oil on Hydatid Cyst Protoscoleces. Vet. Parasitol..

[16] Moazeni M., Saharkhiz M.J., Alavi A.M. (2019). The Lethal Effect of a Nano Emulsion of Satureja Hortensis Essential Oil on Protoscoleces and Germinal Layer of Hydatid Cysts. Iran. J. Parasitol..

[17] Mahmoudvand H., Pakravanan M., Aflatoonian M.R., Khalaf A.K., Niazi M., Mirbadie S.R., Tavakoli Kareshk A., Khatami M. (2019). Efficacy and Safety of *Curcuma longa* Essential Oil to Inactivate Hydatid Cyst Protoscoleces. BMC Complement. Altern. Med..

[18] Mahmoudvand H., Dezaki E.S., Kheirandish F., Ezatpour B., Jahanbakhsh S., Harandi M.F. (2014). Scolicidal Effects of Black Cumin Seed (*Nigella sativa*) Essential Oil on Hydatid Cysts. Korean J. Parasitol..

[19] Mahapatra S.K., Roy S. (2014). Phytopharmacological Approach of Free Radical Scavenging and Anti-Oxidative Potential of Eugenol and Ocimum Gratissimum Linn. Asian Pac. J. Trop. Med..

[20] Charan-Raja M.R., Srinivasan V., Selvaraj S., Mahapatra S.K. (2015). Versatile and Synergistic Potential of Eugenol: A Review. Pharm. Anal. Acta.

[21] Batiha G.E.-S.S., Alkazmi L.M., Wasef L.G., Beshbishy A.M., Nadwa E.H., Rashwan E.K. (2020). Syzygium Aromaticum l. (Myrtaceae): Traditional Uses, Bioactive Chemical Constituents, Pharmacological and Toxicological Activities. Biomolecules.

[22] Xie Y., Yang Z., Cao D., Rong F., Ding H., Zhang D. (2015). Antitermitic and Antifungal Activities of Eugenol and Its Congeners from the Flower Buds of Syzgium Aromaticum (Clove). Ind. Crops Prod..

[23] Kumar P., Singh D.K. (2014). In Vitro Anthelmintic Activity of Allium Sativum, Ferula Asafoetida, Syzygium Aromaticum and Their Active Components against Fasciola Gigantica. J. Biol. Earth Sci..

[24] ElGhannam M., Dar Y., ElMehlawy M.H., Mokhtar F.A., Bakr L. (2023). Eugenol; Effective Anthelmintic Compound against Foodborne Parasite Trichinella Spiralis Muscle Larvae and Adult. Pathogens.

[25] El-kady A.M., Ahmad A.A., Hassan T.M., El-Deek H.E.M.M., Fouad S.S., Althagfan S.S. (2019). Eugenol, a Potential Schistosomicidal Agent with Anti-Inflammatory and Antifibrotic Effects against Schistosoma Mansoni, Induced Liver Pathology. Infect. Drug Resist..

[26] Machado M., Dinis A.M., Salgueiro L., Custódio J.B.A., Cavaleiro C., Sousa M.C. (2011). Anti-Giardia Activity of Syzygium Aromaticum Essential Oil and Eugenol: Effects on Growth, Viability, Adherence and Ultrastructure. Exp. Parasitol..

[27] Santoro G.F., Cardoso M.G., Guimarães L.G.L., Mendonça L.Z., Soares M.J. (2007). Trypanosoma Cruzi: Activity of Essential Oils from *Achillea millefolium* L., *Syzygium aromaticum* L. and *Ocimum basilicum* L. on Epimastigotes and Trypomastigotes. Exp. Parasitol..

[28] Misra P., Kumar A., Khare P., Gupta S., Kumar N., Dube A. (2009). Pro-Apoptotic Effect of the Landrace Bangla Mahoba of Piper Betle on Leishmania Donovani May Be Due to the High Content of Eugenol. J. Med. Microbiol..

[29] Hu Q., Gerhard H., Upadhyaya I., Venkitanarayanan K., Luo Y. (2016). Antimicrobial Eugenol Nanoemulsion Prepared by Gum Arabic and Lecithin and Evaluation of Drying Technologies. Int. J. Biol. Macromol..

[30] Lucia A., Toloza A.C., Fanucce M., Fernández-Peña L., Ortega F., Rubio R.G., Coviella C., Guzmán E. (2020). Nanoemulsions Based on Thymol-Eugenol Mixtures: Characterization, Stability and Larvicidal Activity against Aedes Aegypti. Bull. Insectology.

[31] Maurice M.N., Huseein E.A.M., El-Salahy Monib M.M.M., Alsharif F.M., Namazi N.I., Ahmad A.A. (2021). Evaluation of the Scolicidal Activities of Eugenol Essential Oil and Its Nanoemulsion against Protoscoleces of Hydatid Cysts. PLoS ONE.

[32] Eckert J., Deplazes P. (2004). Biological, Epidemiological, and Clinical Aspects of Echinococcosis, a Zoonosis of Increasing Concern. Clin. Microbiol. Rev..

[33] Rigano R., Profumo E., Ioppolo S., Notargiacomo S., Teggi A., Siracusano A. (1999). Serum Cytokine Detection in the Clinical Follow up of Patients with Cystic Echinococcosis. Clin. Exp. Immunol..

[34] Zhang W., Ross A.G., McManus D.P. (2008). Mechanisms of Immunity in Hydatid Disease: Implications for Vaccine Development. J. Immunol..

[35] Siracusano A., Delunardo F., Teggi A., Ortona E. (2012). Host-Parasite Relationship in Cystic Echinococcosis: An Evolving Story. Clin. Dev. Immunol..

[36] Riganò R., Buttari B., De Falco E., Profumo E., Ortona E., Margutti P., Scottà C., Teggi A., Siracusano A. (2004). Echinococcus Granulosus-Specific T-Cell Lines Derived from Patients at Various Clinical Stages of Cystic Echinococcosis. Parasite Immunol..

[37] Zhu J., Yamane H., Paul W.E. (2010). Differentiation of Effector CD4 T Cell Populations. Annu. Rev. Immunol..

[38] Wang Y., Qu A., Qu A. (2015). Signal Transducer and Activator of Transcription 4 in Liver Diseases. Int. J. Biol. Sci..

[39] Yagi R., Zhu J., Paul W.E. (2011). An Updated View on Transcription Factor GATA3- Mediated Regulation of T h 1 and T h 2 Cell Differentiation. Int. Immunol..

[40] Islamuddin M., Chouhan G., Want Y., Ozbak H.A., Hemeg H.A., Afrin F., Want M.Y., Ozbak H.A., Hemeg H.A., Afrin F. (2016). Immunotherapeutic Potential of Eugenol Emulsion in Experimental Visceral Leishmaniasis. PLoS Negl. Trop. Dis..

[41] Sayed R.E., Barghash S., Alfy N.E., Elnour B.A., Sadek A. (2017). Physiological, Immunological and Histopathological Comparison of Echinococcus Granulosus (G6) Camel Strain By Different Viability Status Using Secondary Cyst Development in Rat. Int. J. Adv. Res..

[42] Labsi M., Soufli I., Khelifi L., Amir Z.C., Touil-Boukoffa C. (2019). A Preventive Effect of the Combination of Albendazole and Pomegranate Peel Aqueous Extract Treatment in Cystic Echinococcosis Mice Model: An Alternative Approach. Acta Trop..

[43] Baqer N.N., Khuder M.H., Amer N. (2014). Antiprotoscolices Effects of Ethanolic Extract of Zingiber Officinale against Echinococcus Granulosus Invitro and Invivo. Int. J. Adv. Res..

[44] Junqueira L.C.U., Bignolas G., Brentani R.R. (1979). Picrosirius Staining plus Polarization Microscopy, a Specific Method for Collagen Detection in Tissue Sections. Histochem. J..

[45] Soliman M., Sadek A.A., Abdelhamid H.N., Hussein K. (2021). Graphene Oxide-Cellulose Nanocomposite Accelerates Skin Wound Healing. Res. Vet. Sci..

[46] Ibrahim A., Soliman M., Kotb S., Ali M.M. (2020). Evaluation of Fish Skin as a Biological Dressing for Metacarpal Wounds in Donkeys. BMC Vet. Res..

[47] Kohansal M.H., Nourian A., Rahimi M.T., Daryani A., Spotin A., Ahmadpour E. (2017). Natural Products Applied against Hydatid Cyst Protoscolices: A Review of Past to Present. Acta Trop..

[48] El-On J. (2003). Benzimidazole Treatment of Cystic Echinococcosis. Acta Trop..

[49] Tagboto S., Townson S. (2001). Antiparasitic Properties of Medicinal Plants and Other Naturally Occurring Products. Adv. Parasitol..

[50] Dawood M.A.O., El Basuini M.F., Zaineldin A.I., Yilmaz S., Hasan M.T., Ahmadifar E., El Asely A.M., Abdel-Latif H.M.R., Alagawany M., Abu-Elala N.M. (2021). Antiparasitic and Antibacterial Functionality of Essential Oils: An Alternative Approach for Sustainable Aquaculture. Pathogens.

[51] Longuespée R., Casadonte R., Kriegsmann M., Wandernoth P., Lisenko K., Mazzucchelli G., Becker M., Kriegsmann J. (2017). Proteomic Investigation of Human Cystic Echinococcosis in the Liver. Mol. Biochem. Parasitol..

[52] Selles S.M.A., Kouidri M., Belhamiti T.B., Ait Amrane A., Benahmed M., Hachemi A. (2020). Main Compounds and In Vitro Effectiveness of Syzygium Aromaticum Essential Oil on Protoscoleces of Hydatid Cyst. Comp. Clin. Path..

[53] Raja M.R.C., Velappan A.B., Chellappan D., Debnath J., Mahapatra S.K. (2017). Eugenol Derived Immunomodulatory Molecules against Visceral Leishmaniasis. Eur. J. Med. Chem..

[54] Raja M.R.C., Kar A., Srinivasan S., Chellappan D., Debnath J., Mahapatra S.K. (2021). Oral Administration of Eugenol Oleate Cures Experimental Visceral Leishmaniasis through Cytokines Abundance. Cytokine.

[55] Junior G.Z., Massago M., Teston A.P.M., Morey A.T., Toledo M.J.O. (2017). Efficacy of Some Essential Oils in Mice Infected with Trypanosoma Cruzi. Trop. J. Pharm. Res..

[56] Pontes K.A., Silva L.S., Santos E.C., Pinheiro A.S., Teixeira D.E., Peruchetti D.B., Silva-Aguiar R.P., Wendt C.H., Miranda K.R., Coelho-de-Souza A.N. (2021). Eugenol Disrupts Plasmodium Falciparum Intracellular Development during the Erythrocytic Cycle and Protects against Cerebral Malaria. Biochim. Biophys. Acta-Gen. Subj..

[57] Ahmad N., Ahmad F.J., Bedi S., Sharma S., Umar S., Ansari M.A. (2019). A Novel Nanoformulation Development of Eugenol and Their Treatment in Inflammation and Periodontitis. Saudi Pharm. J..

[58] Rajabnezhad S., Partoazar A., Mehr S.E., Faridi-majidi R. (2016). Anti-Inflammatory Effects of Eugenol Nanoemulsion as a Topical Delivery System. Pharm. Dev. Technol..

[59] Fabbri J., Maggiore M.A., Pensel P.E., Denegri G.M., Gende L.B., Elissondo M.C. (2016). In Vitro and In Vivo Efficacy of Carvacrol against Echinococcus Granulosus. Acta Trop..

[60] Keyhani A., Kareshk A.T., Oliaei R.T., Mahmoudvand H. (2017). Protoscolicidal Effects and Acute Toxicity of Essential Oil and Methanolic Extract of Cuminum Cyminum Seeds. Marmara Pharm. J..

[61] Moazeni M., Larki S., Saharkhiz M.J., Oryan A., Lari M.A., Alavi A.M. (2014). In Vivo Study of the Efficacy of the Aromatic Water of Zataria Multiflora on Hydatid Cysts. Antimicrob. Agents Chemother..

[62] Mahmoud N., Ayman A., Ibrahim N., Ali N.M., Ibrahim A.N., Ahmed N.S. (2016). Assessment of the Effect of Allium Sativum on Serum Nitric Oxide Level and Hepatic Histopathology in Experimental Cystic Echinococcosis in Mice. J. Parasit. Dis..

[63] Shahnazi M., Azadmehr A., Andalibian A., Hajiaghaee R., Saraei M., Alipour M. (2016). Protoscolicidal and Immunomodulatory Activity of Ziziphora Tenuior Extract and Its Fractions. Asian Pac. J. Trop. Med..

[64] Aryamand S., Khademvatan S., Tappeh K.H., Heshmatian B., Jelodar A. (2019). In Vitro and in Vivo Scolicidal Activities of Holothuria Leucospilota Extract and CEO2 Nanoparticles against Hydatid Cyst. Iran. J. Parasitol..

[65] Al-Kuraishi A.H. (2009). Histopathological Changes of Experimental Hydatidosis in Liver and Spleen of Albino Mice: Age and Sex Effect. J. Fac. Med. Baghdad.

[66] Heymann F., Trautwein C., Tacke F. (2009). Monocytes and Macrophages as Cellular Targets in Liver Fibrosis. Inflamm. Allergy-Drug Targets.

[67] Zhang W., Li J., McManus D.P.D.P. (2003). Concepts in Immunology and Diagnosis of Hydatid Disease. Clin. Microbiol. Rev..

[68] Fathy M., Khalifa E.M.M.A., Fawzy M.A. (2019). Modulation of Inducible Nitric Oxide Synthase Pathway by Eugenol and Telmisartan in Carbon Tetrachloride-Induced Liver Injury in Rats. Life Sci..

[69] Yogalakshmi B., Viswanathan P., Anuradha C.V. (2010). Investigation of Antioxidant, Anti-Inflammatory and DNA-Protective Properties of Eugenol in Thioacetamide-Induced Liver Injury in Rats. Toxicology.

[70] Estevão-Silva C.F., Kummer R., Fachini-Queiroz F.C., Grespan R., de Melo G.A.N., Baroni S., Cuman R.K.N., Bersani-Amado C.A. (2014). Anethole and Eugenol Reduce in Vitro and in Vivo Leukocyte Migration Induced by FMLP, LTB4, and Carrageenan. J. Nat. Med..

[71] Ali S., Prasad R., Mahmood A., Routray I., Shinkafi T.S., Sahin K., Kucuk O. (2014). Eugenol-Rich Fraction of Syzygium Aromaticum (Clove) Reverses Biochemical and Histopathological Changes in Liver Cirrhosis and Inhibits Hepatic Cell Proliferation. J. Cancer Prev..

[72] Beigh A.B., Darzi M.M., Bashir S., Shah A., Shah S.A. (2017). Gross and Histopathological Alterations Associated with Cystic Echinococcosis in Small Ruminants. J. Parasit. Dis..

[73] Ramos G., Ph D., Orduña A., García-yuste M. (2001). Hydatid Cyst of the Lung: Diagnosis and Treatment. World J. Surg..

[74] Magalhães C.B., Casquilho N.V., Machado M.N., Riva D.R., Travassos L.H., Leal-Cardoso J.H., Fortunato R.S., Fa D.S., Faffe D.S., Zin W.A. (2019). The Anti-Inflammatory and Anti-Oxidative Actions of Eugenol Improve Lipopolysaccharide-Induced Lung Injury. Respir. Physiol. Neurobiol..

[75] Huang X., Liu Y., Lu Y., Ma C. (2015). Anti-Inflammatory Effects of Eugenol on Lipopolysaccharide-Induced Inflammatory Reaction in Acute Lung Injury via Regulating Inflammation and Redox Status. Int. Immunopharmacol..

[76] Zhang W., Mcmanus D.P. (2006). Recent Advances in the Immunology and Diagnosis of Echinococcosis. FEMS Immunol. Med. Microbiol..

[77] Rogan M.T. (1998). T-Cell Activity Associated with Secondary Infections and Implanted Cysts of Echinococcus Granulosus in BALB/c Mice. Parasite Immunol..

[78] Rigano R., Profumo E., Ioppolo S., Notargiacomo S., Ortona E., Teggi A., Siracusano A. (1995). Immunological Markers Indicating the Effectiveness of Pharmacological Treatment in Human Hydatid Disease. Clin. Exp. Immunol..

[79] Bayraktar M.R., Mehmet N., Durmaz R. (2005). Th1 and Th2 Inducing Cytokines in Cystic Echinococcosis. Turk. Parazitol. Derg..

[80] Shaheen H.A.A.A.S., El-Ahl S.A.H.S., Aal A.A.A., Raouf A.M.A., Badawi M.A.M. (2020). Human Hydatidosis with Different Therapeutic Modalities: Cellular and Immunological Analysis. Sci. J. Al-Azhar Med. Fac. Girls.

[81] Kar A., Raja M.R.C., Jayaraman A., Srinivasan S., Debnath J., Mahapatra S.K. (2021). Oral Combination of Eugenol Oleate and Miltefosine Induce Immune Response during Experimental Visceral Leishmaniasis through Nitric Oxide Generation with Advanced Cytokine Demand. Cytokine.

[82] Mourglia-Ettlin G., Marqués J.M., Chabalgoity J.A., Dematteis S. (2011). Early Peritoneal Immune Response during Echinococcus Granulosus Establishment Displays a Biphasic Behavior. PLoS Negl. Trop. Dis..

[83] Hernández A., O’Connor J.E., Mir A. (1999). Phenotypic Analysis of Peripheral Lymphocyte Subpopulations in Hydatid Patients. Parasitol. Res..

[84] Vishteh A., Thomas I., Imamura T. (1986). Eugenol Modulation of the Immune Response in Mice. Immunopharmacology.

[85] Ma X.M., Bao G.S., Wan J.M., Liao D.J., Yin S.F., Meng X.Q., Zhou G.K., Lu X.M., Li H.Y. (2007). Therapeutic Effects of Sophora Moorcroftiana Alkaloids in Combination with Albendazole in Mice Experimentally Infected with Protoscolices of Echinococcus Granulosus. Braz. J. Med. Biol. Res..

[86] Kilkenny C., Browne W., Cuthill I.C., Emerson M., Altman D.G., Group N.R.G.W. (2010). Animal Research: Reporting in Vivo Experiments: The ARRIVE Guidelines. Br. J. Pharmacol..

