# Supplementary material for: Eugenol Essential Oil and Nanoemulsion as Antihydatic Agents with Antifibrotic and Immunomodulatory Effects in Cystic Echinococcosis
Source: Trop Med Infect Dis. 2023 Apr 27;8(5):253. doi: 10.3390/tropicalmed8050253 (PMC10223691; doi:10.3390/tropicalmed8050253)
Supplement: Supplementary file 1 [file tropicalmed-08-00253-s001.zip › tropicalmed-2302351-supplementary.pdf]

Supplementary materials

Table S1 : Eugenol and eugenol nanoemulsion effects on rat's liver weight.

GROUPS	Liver average Wt. (gm)	*P*-value**		
**1. Unninfected Control**	5.58 ± 0.3	1 vs 2<0.001	2 vs 7<0.001	4 vs 6<0.001
**2. Infected Control CE**	15.11 ± 0.9	1 vs 3=0.005	2 vs 8<0.001	4 vs 7=0.001
**3. Infected treated Eug-NE**	7.04 ± 0.6	1 vs 4=0.002	3 vs 4=0.049	4 vs 8<0.001
**4. Infected treated Eug**	7.79 ± 0.5	1 vs 5<0.001	3 vs 5=0.002	5 vs 6<0.001
**5. Infected treated ABZ.**	8.80 ± 0.5	2 vs 3<0.001	3 vs 6=0.008	5 vs 7<0.001
**6. Uninfected treated Eug**	5.96 ± 0.3	2 vs 4<0.001	3 vs 7=0.010	5 vs 8<0.001
**7. Uninfected treated Eug-NE**	5.85 ± 0.3	2 vs 5<0.001	3 vs 8=0.004	
**8. Uninfected treated ABZ.**	5.87 ± 0.4	2 vs 6<0.001	4 vs 5=0.015	
P*-value	**< 0.001**			

*One Way ANOVA **Post-hoc test *P*-value< 0.05 was considered significant.

Table S2 : Eugenol and eugenol nanoemulsion effects on rat's liver hypertrophy indicator.

GROUPS	Liver hypertrophy indicator (%)	*P*-value**		
**1. Uninfected Control**	29.25 ± 1.4	1 vs 2<0.001	2 vs 7<0.001	4 vs 6<0.001
**2. Infected Control CE**	78.70 ± 4.8	1 vs 3=0.004	2 vs 8<0.001	4 vs 7=0.001
**3. Infected treated Eug-NE**	35.57 ± 3.0	1 vs 4=0.001	3 vs 4=0.042	4 vs 8<0.001
**4. Infected treated Eug**	39.55 ± 2.6	1 vs 5<0.001	3 vs 5=0.001	5 vs 6<0.001
**5. Infected treated ABZ.**	45.14 ± 2.7	2 vs 3<0.001	3 vs 6=0.006	5 vs 7<0.001
**6. Uninfected treated Eug**	29.80 ± 1.5	2 vs 4<0.001	3 vs 7=0.008	5 vs 8<0.001
**7. Uninfected treated Eug-NE**	29.24 ± 1.7	2 vs 5<0.001	3 vs 8=0.003	
**8. Uninfected treated ABZ.**	29.34 ± 1.9	2 vs 6<0.001	4 vs 5=0.011	
P*-value	**< 0.001**			

*One Way ANOVA **Post-hoc test *P*-value< 0.05 was considered significant.

Table S3 : Eugenol and eugenol nanoemulsion effects on rat's lung weight.

GROUPS	Lung average Wt. (gm)	*P*-value**		
**1. Uninfected Control**	1.49 ± 0.1	1 vs 2<0.001	2 vs 7<0.001	4 vs 7<0.001
**2. Infected Control CE**	3.57 ± 0.3	1 vs 3<0.001	2 vs 8<0.001	4 vs 8<0.001
**3. Infected treated Eug-NE**	1.99 ± 0.1	1 vs 4<0.001	3 vs 4=0.005	5 vs 6<0.001
**4. Infected treated Eug**	2.50 ± 0.2	1 vs 5<0.001	3 vs 5=0.001	5 vs 7<0.001
**5. Infected treated ABZ.**	2.73 ± 0.2	2 vs 3<0.001	3 vs 6<0.001	5 vs 8<0.001
**6. Uninfected treated Eug**	1.45 ± 0.1	2 vs 4=0.003	3 vs 7<0.001	
**7. Uninfected treated Eug-NE**	1.37 ± 0.1	2 vs 5=0.004	3 vs 8<0.001	
**8. Uninfected treated ABZ.**	1.31 ± 0.1	2 vs 6<0.001	4 vs 6<0.001	
P*-value	< 0.001			

*One Way ANOVA **Post-hoc test *P*-value< 0.05 was considered significant.

Table S4 : Eugenol and eugenol nanoemulsion effects on rat's lung hypertrophy indicator.

GROUPS	Lung hypertrophy indicator (%)	*P*-value**		
**1. Uninfected Control**	7.46 ± 0.4	1 vs 2<0.001	2 vs 7<0.001	4 vs 7<0.001
**2. Infected Control CE**	17.98 ± 1.4	1 vs 3<0.001	2 vs 8<0.001	4 vs 8<0.001
**3. Infected treated Eug-NE**	9.97 ± 0.7	1 vs 4<0.001	3 vs 4=0.005	5 vs 6<0.001
**4. Infected treated Eug**	12.52 ± 1.1	1 vs 5<0.001	3 vs 5=0.001	5 vs 7<0.001
**5. Infected treated ABZ.**	13.79 ± 1.1	2 vs 3<0.001	3 vs 6<0.001	5 vs 8<0.001
**6. Uninfected treated Eug**	7.27 ± 0.6	2 vs 4=0.002	3 vs 7<0.001	
**7. Uninfected treated Eug-NE**	6.84 ± 0.6	2 vs 5=0.004	3 vs 8<0.001	
**8. Uninfected treated ABZ.**	6.53 ± 0.4	2 vs 6<0.001	4 vs 6<0.001	
P*-value	= 0.004			

*One Way ANOVA **Post-hoc test *P*-value< 0.05 was considered significant.
